# Default-Mode-Like Network Activation in Awake Rodents

**DOI:** 10.1371/journal.pone.0027839

**Published:** 2011-11-18

**Authors:** Jaymin Upadhyay, Scott J. Baker, Prasant Chandran, Loan Miller, Younglim Lee, Gerard J. Marek, Unal Sakoglu, Chih-Liang Chin, Feng Luo, Gerard B. Fox, Mark Day

**Affiliations:** 1 Translational Sciences, Advanced Technology, Global Pharmaceutical Research and Development, Abbott Laboratories, Abbott Park, Illinois, United States of America; 2 Neuroscience Discovery, Global Pharmaceutical Research and Development, Abbott Laboratories, Abbott Park, Illinois, United States of America; 3 Neuroscience Development, Global Pharmaceutical Research and Development, Abbott Laboratories, Abbott Park, Illinois, United States of America; Cuban Neuroscience Center, Cuba

## Abstract

During wakefulness and in absence of performing tasks or sensory processing, the default-mode network (DMN), an intrinsic central nervous system (CNS) network, is in an active state. Non-human primate and human CNS imaging studies have identified the DMN in these two species. Clinical imaging studies have shown that the pattern of activity within the DMN is often modulated in various disease states (e.g., Alzheimer's, schizophrenia or chronic pain). However, whether the DMN exists in awake rodents has not been characterized. The current data provides evidence that awake rodents also possess ‘DMN-like’ functional connectivity, but only subsequent to habituation to what is initially a novel magnetic resonance imaging (MRI) environment as well as physical restraint. Specifically, the habituation process spanned across four separate scanning sessions (Day 2, 4, 6 and 8). At Day 8, significant (p<0.05) functional connectivity was observed amongst structures such as the anterior cingulate (seed region), retrosplenial, parietal, and hippocampal cortices. Prior to habituation (Day 2), functional connectivity was only detected (p<0.05) amongst CNS structures known to mediate anxiety (i.e., anterior cingulate (seed region), posterior hypothalamic area, amygdala and parabracial nucleus). In relating functional connectivity between cingulate-default-mode and cingulate-anxiety structures across Days 2-8, a significant inverse relationship (r = −0.65, p = 0.0004) was observed between these two functional interactions such that increased cingulate-DMN connectivity corresponded to decreased cingulate anxiety network connectivity. This investigation demonstrates that the cingulate is an important component of both the rodent DMN-like and anxiety networks.

## Introduction

One fundamental property of the CNS is its functional organization. This is evident during the resting-state, where neuronal structures spontaneously and coherently fluctuate to form functionally specific brain networks [1], [2], [3], [4]. Given the metabolic demand necessary to support such spontaneous and coherent fluctuations, CNS networks with distinct spatial-temporal properties can be identified and characterized by methods such as resting-state, blood-oxygenated level dependent (BOLD) functional MRI (fMRI) or [^18^F] FDG-Positron Emission Tomography [1], [2], [3], [4], [5]. The DMN, an intrinsic resting-state CNS network, is fully active during wakefulness and in the absence of purposeful cognitive task performance or processing of external sensory cues [1]. To date, the DMN has been identified in humans [1], [2], [3], [4] and non-human primates [6], [7], and is primarily believed to be specific to such higher-order species.

In this study, we aimed to identify functional connectivity amongst brain structures that define the human and non-human primate DMN in lower-order species (i.e., rats) using awake resting-state, BOLD fMRI. It has been shown that the use of anesthesia can perturb the coherence strength within resting-state CNS networks [6], [8]. Thus, to eliminate anesthesia-related confounds, resting-state BOLD fMRI data was collected in awake rats.

The anterior segment of the cingulate cortex has been shown to be a central hub of the DMN in humans as well as in non-human primates. To determine if rodent DMN activity exists, the anterior cingulate cortex was used as a seed region in functional connectivity analysis. We hypothesized that as animals became habituated to the MRI scanning environment (also determined by factors such as respiratory rate during the scanning session), enhanced coherence or functional connectivity would be observed amongst brain structures that define the DMN in non-human primates and humans (e.g., anterior cingulate, parietal and retrosplenial cortices). Given the role of the anterior cingulate cortex in other CNS processes, such as mediating negative affect or pain [9], anterior cingulate-based functional connectivity analysis could also yield information regarding modulation of activity in other networks during the habituation process.

## Materials and Methods

### Animals

#### Ethics Statement

All studies were conducted in accordance with Institutional Animal Care and Use Committee (IACUC) guidelines at Abbott Laboratories as well as the National Institutes of Health Guide for Care and Use of Laboratory Animals guidelines. The facilities at Abbott Laboratories are accredited by the Association for the Assessment and Accreditation of Laboratory Animal Care (AAALAC).

Adult male Long-Evans rats (Charles River, Portage, MI) were used (n = 8; 225∼250 g at start). Rats were group-housed (2 per cage) in temperature-controlled (22°–24°C) rooms and maintained on a 12:12 light:dark cycle with lights on at 6:00 am. Rat chow and water were provided *ad libitum*.

In the current study, each animal was subjected to three distinct experimental procedures; (i) 5-day experimenter handling period (Days -7 to -2), (ii) 2-day restrainer training session (Days -2 to -1) and (iii) 8-day awake imaging period (Days 1 to 8).

### Pre-MRI Acclimation Training

#### 5-day experimenter handling period (Days -7 to -2)

For 5 consecutive days each animal was handled individually for a period of 15-20 minutes in order to establish a familiarity both with being held and with the experimenter. Handling included hand-to-hand walking and gentle petting to ensure that animals were familiar with being held at a level above normal handling requirements for normal cage changing. This handling was preformed prior to any exposure to the MRI awake restrainer. Plasma was collected from the 8 subjects under light anesthesia (3% isoflurane) using the tail-nick method [10] on Day 1 an hour before the first handling and on day 5 immediately following the final handling. A slight, but insignificant (t_7_ = -1.45, p = 0.19) decrease in plasma corticosterone was detected between Day 1 (378.43±68.66 ng/mL) and Day 5 (287.05±27.38 ng/mL) of handling.

#### 2-day restrainer training session (Days -2 to -1)

After the 5^th^ day of handling, rats were exposed to the identical type of restrainer (Insight Neuroimaging Systems, Worcester MA) as was used during fMRI scanning for 10 and 30 minute periods on consecutive days. The restrainer consists of a long plastic tube for the rat body that fits into a headgear apparatus that secures the animals head using a bite-bar and halo headgear. For training sessions the rat in the restrainer is placed in a behavioral acclimation chamber with a speaker system inside that mimics the audible levels of a MRI scan. Light anesthesia (3% isoflurane) was administered for the ∼3 minute period required to secure animals in the restrainer. Animals were awake within 10–15 minutes after isoflurane administration and prior to the awake imaging simulation sessions. Plasma was collected immediately following the restrainer exposures. An insignificant increase (t_7_ = −1.29, p = 0.24) in plasma corticosterone was detected between Day 1 (350.20±37.07 ng/mL) and Day 2 (415.77±24.28 ng/mL) of restrainer training. The slight elevation in corticosterone levels between Day -2 and Day -1 was believed to result from the increased time spent within the MRI simulator (10 mins vs. 30 mins). In a comparison of corticosterone levels between the last day of handling with each restrainer training day, a significant increase was detected between handling and the second restrainer training session (t_7_ = -3.04, p = 0.019), but not the first restrainer training session (t_7_ = -2.33, p = 0.052). The pre-MRI acclimation training and awake resting-state fMRI scanning were adapted from previous investigations [11].

### Awake Resting-State BOLD fMRI

#### 8-day awake imaging period (Days 1 to 8)

Following the final restrainer exposure, animals were given a 48-hour recovery period before beginning fMRI habituation scans. FMRI data were collected over an 8-day period, Day 1, 2, 4, 6 and 8. Animals were secured in the restrainer as described previously. Given the MRI setup and awake MRI restrainer, it was not possible to collect corticosterone levels while the animals were inside the MRI. All scanning was performed on a 7T Bruker Biospec (Karlsruhe, Germany) MRI magnet. During MRI data collection, the animals remained within a dark environment (lights were turned off within the MRI scanner room. **High Resolution Structural MRI parameters:** T2-weighted RARE, echo time  =  75 milliseconds, time of repetition  =  3300 milliseconds, Resolution 0.5×0.5×1.25 millimeters^3^, 14 coronal slices. **fMRI parameters:** Single-shot gradient echo planar imaging, echo time  =  11.11 milliseconds, time of repetition  =  2000 milliseconds, repetitions  =  900, Resolution 0.5×0.5×1.25 millimeters^3^ 14 coronal slices. The respiratory rate was monitored and recorded during each scan (SAM32-PC: SA Instruments). During the 30-minute resting-state BOLD fMRI scan, respiratory rates were measured at 0, 10, 20 and 30-minute time points.

### Resting-State BOLD fMRI Analysis

All resting-state, BOLD fMRI data analysis was performed using FMRIB Software Library (FSL 4.1) (www.fmrib.ox.ac.uk/fsl). The initial preprocessing procedures and single-subject general linear model (GLM) analysis of fMRI data was carried out with FSL's FMRI Expert Analysis Tool (FEAT) with local autocorrelation correction. Initially, a 4-minute block was segmented at the 20-minute time point of the fMRI dataset. The 4-minute block was chosen based on the fact that minimal motion (See Results) was detected in this time interval, yet was long enough to characterize functional connectivity. The following processing steps were performed to the remaining 120 volumes: 1) Motion correction with FMRIB's Linear Motion Correction tool (MCFLIRT), 2) Manual segmentation of the brain from the skull and surrounding tissue 3) Spatial smoothing with a 1 mm FWHM Gaussian spatial filter, 4) Band-pass filtered between 0.01 and 0.1 Hz; thus removing the linear drift artifact and high frequency noise, 5) Co-registration to an in-house rat atlas template [12]. Warping of individual datasets to the rat atlas was performed with FMRIB's Linear Image Registration Tool (FLIRT) with a 12 degree-of-freedom affine transformation. During the co-registration procedure, a lower resolution T2-weighted RARE anatomical image was used as an intermediary volume between resting-state fMRI and an in-house, high-resolution rat anatomical template.

For single-subject seed region functional connectivity analysis, subject-specific time courses from the anterior cingulate cortex, white matter and cerebral spinal fluid (CSF) timecourses were extracted from each subject. In single-subject GLM analysis, the anterior cingulate cortex time course was used as the main explanatory variable, while subject-specific time courses from the white matter and CSF were used as confound EVs. Within group (i.e., Day2 and Day 8) and between group (Day 2 vs. Day 8) analysis was also performed with FEAT. Group-level analysis was performed using FSL's mixed-effects FLAME 1 method [13]. Cluster size correction for all activation maps were also performed [14]. All group-level and cluster size corrected statistical maps obtained from the within group and between group mixed- effects analysis were superimposed and depicted upon an in-house, high-resolution rat anatomical template. Single-subject and group-level functional connectivity changes were quantified by extraction of parameter estimates from atlas-defined structures. Parameter estimates in the context of this GLM-based functional connectivity analysis determined the extent to which the features of a voxel-based timecourse is similar in terms of shape to the explanatory variable (i.e., average timecourse of the anterior cingulate). The lower the parameter estimates, the poorer the fit of the voxel timecourses by the seed region timecourse. A single factor ANOVA analysis was performed to identify overall inter-scan day differences. To gain additional insight and specificity, we performed specific comparisons between each scanning day. 2-tailed, t-test (α = 0.05) were used for all other statistical analysis and quantification in this study, for example, respiratory rates, head displacement and volume of interest (VOI) summary statistics. For each t-test the p-value (p), t-statistic (t) and degrees of freedom (DOF) are reported.

## Results

**Figure 1:**
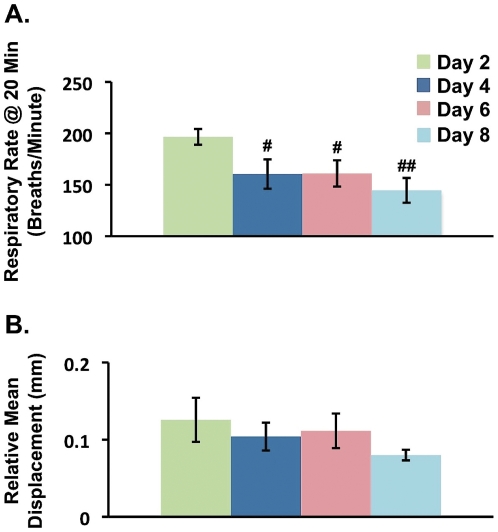
Respiratory Rates and Head Displacement During Awake Resting-State fMRI for Days 2-8. **A.** Respiratory rates significantly decreased between Day 2 and every other scanning session (Days 4-8) where the lowest respiratory rate was measured at Day 8. Respiratory rates between Day 2 and each of the other scanning days (Days 4, 6 and 8) were statistically compared using a 2-tailed unpaired, t-tests. (**Day 4**: t_10_ = 2.42, p = 0.036; **Day 6**: t_12_ = 2.52, p = 0.027; **Day 8**: t_12_ = 3.64, p = 0.027) The ANOVA test revealed significant between day differences for respiratory rate (F_3,22_ = 3.99, p = 0.021), but not for head displacement (F_3,22_ = 0.88, p = 0.47). **B.** Head displacement was less than the size of the imaging voxel for all scanning sessions, but did not significantly differ between Days 2-8. The displacement is relative to a reference volume (Volume 60 of 120). Respiratory rates and head displacement between Day 2 and each of the other scanning days (Days 4, 6 and 8) were statistically compared using a 2-tailed unpaired, t-tests (**Day 4**: t_10_ = 0.58, p = 0.57; **Day 6**: t_12_ = 0.39, p = 0.70; **Day 8**: t_12_ = 1.55, p = 0.15). Error bars represent s.e.m. Day 2 (N = 7); Day 4 (N = 5); Day 6 (N = 7); Day 8 (N = 7). # p<0.05 and ## p<0.005.

During the 30-minute resting-state BOLD fMRI scan, respiratory rates were measured at 0, 10, 20 and 30 minutes. We aimed to characterize resting-state activity during a 4-minute time interval in the awake rat during each scanning session from Days 1-8. Data from all subjects was not usable due to head motion artifact. During the 4-minute time interval, the mean head displacement was required to be less than the in-plane voxel dimension (Resolution: 0.5×0.5×1.25 mm^3^) in order to avoid head motion artifact. At Day 1, frequent head movement (greater than 2-3 x imaging voxel) occurred in all animals and across the 30 minutes. Thus, data from Day 1 was not reliable or usable despite application of motion correction procedures. For Days 2, 4, 6, and 8, data was also not usable due to motion artifact for 1, 3, 1 and 1 rats, respectively. For subjects with fMRI data void of motion artifact, the 4-minute intervals of imaging data as well as single-time-point respiratory rates collected every 10 minutes (0, 10, 20, 30 minute time points) were analyzed. At the 20-minute time point, we observed a decreasing trend in respiratory rate between Day 2 and Day 8 (**Figure 1A**). Baseline respiratory rate for the adult male rat can range between 70-115 breaths/minute (www.criver.com). Importantly, head displacement across the 4 minutes of resting-state fMRI data and at the 20-minute time point was less than the imaging voxel dimension (**Figure 1B**). The group-level (mean ± s.e.m.) respiratory rate and head displacement at the 20-minute time point were lowest at Day 8.

**Figure 2:**
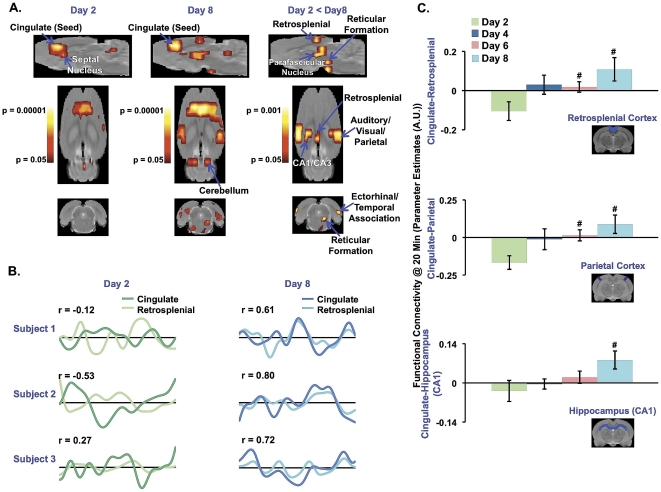
Presence of DMN-like Activity in Awake Rodents. **A.** At Day 2, seed region (anterior cingulate cortex) functional connectivity analysis did not yield CNS circuitry indicative of the DMN, but rather circuitry mediating anxiety (**Figure 3**). CNS circuitry reflective of the DMN emerged at Day 8, when animals were more habituated to the MRI environment. All modeling and estimation of within-group (Day 2 and Day 8) and between-group (Day 2 < Day 8) functional connectivity with the anterior cingulate cortex was performed using mixed-effects analysis. Cluster size correction for all activation maps was also performed. All statistical maps are superimposed upon an in-house, high-resolution rat anatomical template. **B.** Time courses demonstrate increased coherence between cingulate and retrosplenial cortices at Day 8. **C.** An increasing trend in functional connectivity between the cinguate cortex and three other DMN structures was observed between Days 2-8. The ANOVA test for the three functional interactions quantified in **C.** An increasing trend in functional connectivity between the cinguate cortex and three other DMN structures was observed between Days 2-8. The ANOVA test for the three functional interactions quantified in **C.** were significant for cingulate-retrospenial (F_3,22_ = 3.69, p = 0.027) and cingulate-parietal (F_3,22_ = 4.44, p = 0.014) but not for cingulate-CA1 (F_3,22_ = 2.57, p = 0.080). Two-tailed, t-test were also implemented for specific pairwise comparisons (cingulate-retrosplenial- **Day 4**: t_10_ = -1.92, p = 0.084; **Day 6**: t_12_ = -2.26, p = 0.043; **Day 8**: t_12_ = -2.79, p = 0.016). (cingulate-parietal- **Day 4**: t_10_ = -1.96, p = 0.078; **Day 6**: t_12_ = -3.09, p = 0.0093; **Day 8**: t_12_ = -3.34, p = 0.0059). (cingulate-CA1- **Day 4**: t_10_ = -0.51, p = 0.62; **Day 6**: t_12_ = -1.13, p = 0.28; **Day 8**: t_12_ = -2.23, p = 0.045). All error bars represent s.e.m. Day 2 (N = 7); Day 4 (N = 5); Day 6 (N = 7); Day 8 (N = 7). # p<0.05.

Mixed-effects group analysis showed robust and significant enhancement in DMN-like functional connectivity at Day 8 (**Figure 2**). In **Figure 2A**, the within-group anterior cingulate-based functional connectivity maps are shown for Day 2 and Day 8 as well as the between-group functional connectivity contrast map (Day 2 < Day 8). For Day 2, the anterior cingulate cortex was not significantly functionally connected to other DMN structures. In contrast, within-group functional connectivity at Day 8 as well as the group comparison (Day 2 < Day 8) demonstrated functional connectivity between the anterior cingulate cortex and key structures previous defined in the non-human primate and human DMN (i.e., retrosplenial cortex, parietal cortex, temporal association cortex and hippocampus) [1], [4]. Using a 12-parameter affine transformation, the subject-specific, 4-minute, resting-state fMRI datasets were co-registered to an in-house rat template brain, where the cingulate and retrosplenial cortices were defined. This co-registration procedure enabled single subject time courses to be extracted from both structures. In **Figure 2B**, cingulate and retrosplenial time courses extracted from three representative subjects and from Days 2 and 8 are depicted. For all three subjects, an increase in correlation (r) from Day 2 to Day 8 between the cingulate and retrosplenial cortices further demonstrated enhanced functional connectivity or coherence between two DMN structures at Day 8 in comparison to Day 2 (**Figure 2B**). Moreover, an increasing trend in functional connectivity amongst the anterior cingulate cortex and atlas-defined VOIs such as the retrosplenial cortex, parietal cortex and hippocampus can be observed across Days 2-8 (**Figure 2C**). Functional connectivity at Day 4 and Day 6 with respect to Day 2 was also characterized. However, the change in functional connectivity within this network at Day 4 and 6 relative to Day 2 was not as robust in comparison to Day 8. Thus, at Day 8, the DMN-like functional connectivity was strongest, while the respiratory rate and head displacement were lowest (**Figure 1**). However, to ensure that the increasing trend in functional connectivity between Days 2-8 observed in Figure 2C were not driven by changes in global physiology (i.e., respiratory rate) or degree of head displacement, a correlation analysis was performed. The correlation analysis did not reveal any significant correlations or inverse correlations for the three functional interactions (cingulate-retrosplenial, cingulate-parietal and cingulate-CA1). For example, a significant relationship for the cingulate-retrosplenial interaction with respiratory rates (R = -0.095, P = 0.66) or head motion (R = −0.22, p = 0.28) was not detected.

**Figure 3:**
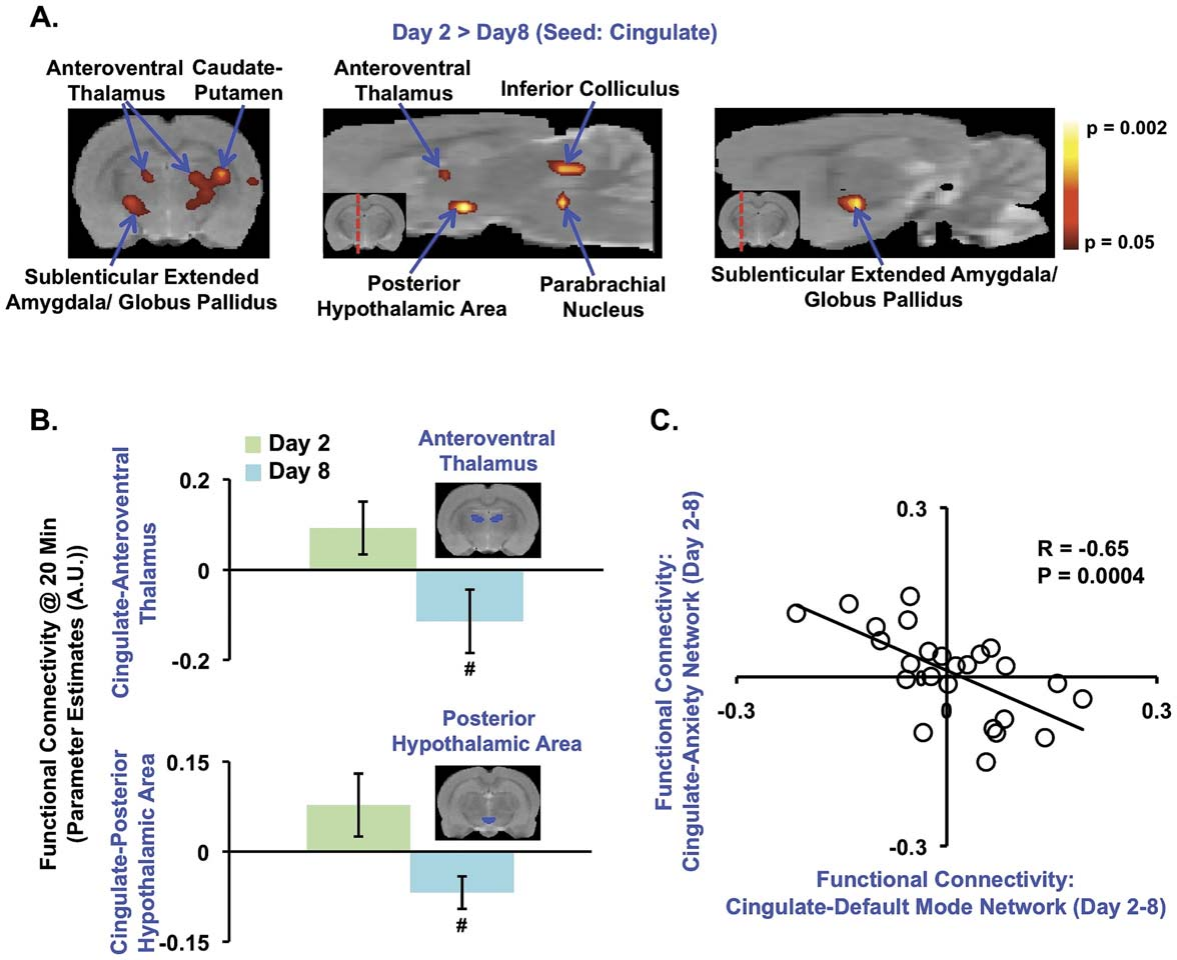
Functional Connectivity in the Anxiety Network in Non-habituated, Awake Rats. **A-B.** Functional connectivity amongst the anterior cingulate cortex and brain regions implicated in anxiety was significantly greater during the initial scanning session at Day 2. All statistical maps are superimposed upon an in-house, high-resolution rat anatomical template. **B.** Functional connectivity at Day 2 and Day 8 is quantified between the cingulate and anteroventral thalamus, and also with the posterior hypothalamic area. The ANOVA test yielded insignificant results for the cingulate-anteroventral thalamus (F_3,22_ = 2.49, p = 0.087) and cingulate-posterior hypothalamic area (F_3,22_ = 1.37, p = 0.27) functional interactions. Two-tailed, t-test did reveal significant differences particularly between Day 2 and Day 8 (cingulate-anteroventral thalamus- **Day 4**: t_10_ = 1.91, p = 0.09; **Day 6**: t_12_ = 1.31, p = 0.22; **Day 8**: t_12_ = 2.26, p = 0.043). (cingulate-posterior hypothalamic area- **Day 4**: t_10_ = 1.07, p = 0.30; **Day 6**: t_12_ = 0.52, p = 0.61; **Day 8**: t_12_ = 2.74, p = 0.018). All error bars represent s.e.m. Day 2 (N = 7); Day 4 (N = 5); Day 6 (N = 7); Day 8 (N = 7). # p<0.05 **C.** A significant inverse correlation was detected between the cingulate-default mode and cingulate-anxiety network structures. One statistical outlier (Z = 3.76) was detected using Grubb's test for outlier detection, and not included in correlation analysis. For N = 26, Z-critical  =  2.84 (p<0.05).

At Day 2, functional connectivity between the anterior cingulate cortex and brain structures such as the posterior hypothalamic area, amygdala, parabrachial nucleus and anteroventral thalamus was greater in comparison to Day 8 (**Figure 3**). Structures demarcated in **Figure 3A** are known to mediate anxiety [15], [16], [17], [18], [19]. In **Figure 3B**, functional connectivity between the anterior cingulate cortex and regions such as the anteroventral thalamus and posterior hypothalamic area further demonstrate the greater functional connectivity in this network at Day 2 in comparison to Day 8. Similar to functional connectivity changes observed in the DMN across Days 2-8, functional connectivity at Day 4 or Day 6 relative to Day 2 was not as robust or significant in comparison to Day 8. Moreover, from Days 2-8, a decreasing trend in functional connectivity was observed between the anterior cingulate cortex and the two regions quantified in **Figure 3B**. Moreover, the high functional connectivity in this anxiety network at Day 2 coincided with the higher respiratory rates and head displacement (**Figure 1**). In relating the functional connectivity (Days 2-8) between the cingulate and anteroventral thalamus with head displacement (Days 2-8), we observed a significant correlation (R = 0.42, p = 0.03). This specific correlation suggests that the greater the head displacement, the greater the functional connectivity measured between the cingulate and anteroventral thalamus. A significant correlation involving respiratory rates was not observed (R = 0.17, p = 0.40). Moreover, functional connectivity between cingulate and posterior hypothalamic area was not correlated or inversely correlated with either respiratory rates (R = 0.20, p = 0.33) or head displacement (R = 0.24, p = 0.24).

In order to characterize changes occurring in anterior cingulate-based functional connectivity between Days 2-8 and across all subject, a correlation analysis was performed using parameter estimates extracted from DMN structures as well as structures mediating anxiety. Here, a significant inverse correlation in anterior cingulate-based functional connectivity across Days 2-8 was observed between structures of the DMN (i.e., retrospenial, parietal and hippocampus (CA1+CA3), etc) and structures mediating anxiety (i.e., posterior hypothalamic area, sublenticular extended amygdala, globus pallidus, etc) (**Figure 3C**).

**Figure 4:**
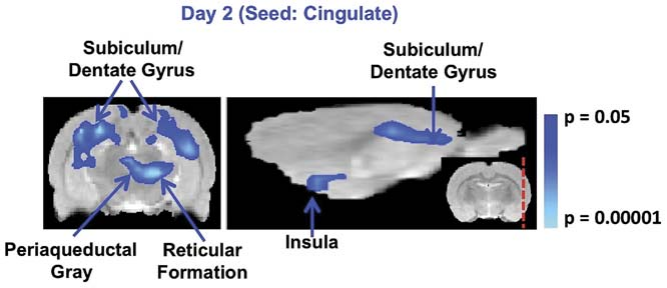
At Day 2, the within group, functional connectivity analysis demonstrated a significant de-coherence between the cingulate and insula cortex as well as the hippocampal formation (dentate gyrus, subiculum) and periaqueductal gray. Significant de-coherences between the cingulate and components of the task positive network such as the motor cortex were not observed.

At Day 2, the DMN was observed to be in an inactive state as a result of the highly anxious state present in rodents. It is possible that this highly anxious state may have similar effects on functional connectivity as conventional task performance or presentation of sensory stimuli. Thus, if an anxious state has similar consequences as a task or sensory stimulation, a de-coherence may be observed between a DMN structure (anterior cingulate) and components of the task positive network (insula, supplementary motor or cortices). The within group, functional connectivity analysis at Day 2 demonstrated a de-coherence between the cingulate and insula cortex as well as the hippocampal formation (dentate gyrus, subiculum) and periaqueductal gray (**Figure 4**). However, significant de-coherences between the cingulate and structures such as the motor cortex were not observed.

## Discussion

In the current study, an experimental imaging protocol was implemented where awake rodents were habituated to the MRI environment over the course of an 8-day period. Using this functional imaging paradigm and anterior cingulate-based functional connectivity analysis, DMN-like functional connectivity in awake rodents was identified and characterized. Coherent resting-state activity amongst CNS structures such as the anterior cingulate, retrosplenial, parietal and hippocampal regions was only observed once awake rodents were habituated to the MRI environment (**Figure 2**). These structures have been previously shown to encompass the DMN in humans [1], [2], [3], [4] as well as non-human primates [6], [7]. Both imaging and non-imaging (**Figure 1**) data indicate that at Day 8, animals were in a ‘resting-state’ relative to earlier scanning periods. It is noted that at Day 6, a significant increase in functional connectivity relative to Day 2 was indeed observed between two structures known to be part of the human and non-human DMN network; for example, the anterior cingulate and retrosplenial cortices (Day 6: p = 0.043). However, at Day 6 the increase functional connectivity was not significant for all functional connections (i,e., anterior cingulate-hippocampus (p = 0.282)). The results presented herein are in accord with previous rodent imaging studies where ‘DMN-like’ activity had been suggested, but not confirmed [20], [21]. In conjunction with previous human and non-human primate studies, these results demonstrate the preservation of DMN-like activity across rodents, non-human primates and humans.

A number of clinical imaging studies have reported altered connectivity or coherence in the DMN in CNS diseases such as Alzheimer's [22], schizophrenia [23] and chronic pain [24], where the modulation of DMN connectivity is distinct between one CNS disease and another. For example, while a decrease in functional connectivity between the posterior cingulate (retrosplenial cortex) and hippocampus has been observed in Alzheimer's patients [22], increased connectivity amongst DMN structures is observed in schizophrenic patients as well as their relatives [25]. Thus, as demonstrated by Seeley et al, alterations in connectivity within large-scale CNS networks, including the DMN, can be used to phenotype CNS diseases [26]. Given that preclinical investigation often include naïve rodents as well as rodent models of CNS diseases, the ability to identify a rodent DMN as well as other CNS circuits [20], [21] may offer a novel and objective means to assess the degree of commonality amongst clinical CNS diseases and the associated preclinical disease model. For example, are the disruptions in DMN connectivity observed in chronic back pain patients [24], recapitulated in preclinical pain models such as the spinal nerve ligation model? If there is consistency in CNS connectivity changes between clinical and preclinical populations, the evaluation of CNS connectivity potentially can offer a translatable mean and measure in pharmacological investigation. Moreover, non-anesthetized functional imaging in rodents may be particularly important when characterizing the impact of pharmacological compounds on CNS circuits when considering the potential confounding effects of anesthesia-compound interactions.

At Day 2, significant functional connectivity was observed between the anterior cingulate and structures that mediate anxiety [15], [16], [17], [18] rather than those that comprise of the DMN (**Figure 3A-3B**). Specifically, anxiety mediating CNS structures such as the posterior hypothalamic area (hypothalamic hub regulating anxiety [17]) as well as the globus pallidus [27] were both functionally connected to the anterior cingulate only at Day 2. The anteroventral thalamus has reciprocal connection with the anterior cingulate cortex and is implicated in mediating learning, memory and attention [28], [29]. Together with the mediodorsal thalamus, the anteroventral thalamus is interconnected with both subcortical and cortical limbic structures (e.g., hypothalamus and amygdala) known to mediate emotional responses [30], [31], [32]. Brainstem structures such as the parabrachial nucleus have previously been implicated in anxiety responses as well as anxiety disorders [33]. Interestingly, ascending projections to the anterior cingulate cortex and descending projections to the parabrachial nucleus innervate the posterior hypothalamic area [18]. Moreover, it was observed that the greater the head displacement, the greater the functional connectivity measured between the cingulate and anteroventral thalamus. This correlation may further suggest that anxiety network functional connectivity diminished between Days 2-8 as animals became calmer in the scanner.

To additionally characterize anterior cingulate-based functional connectivity dynamics across Days 2-8 and for all animals, the connectivity strength for the cingulate-DMN functional interaction was related with connectivity strength with the cingulate-anxiety network functional interaction (**Figure 3C**). The inverse correlation detected between functional interactions further demonstrated the transition amongst the DMN-like and anxiety networks in the current study.

Humans and non-human primate functional connectivity studies have demonstrated that during a task positive network is inversely correlated with the DMN [34], [35], [6]. It may be considered that when an awake rodent is in a highly anxious state such as at Day 2 of scanning, a task-like state may be present. Measuring which CNS structures have de-coherence with the anterior cingulate may identify a task positive network. At Day 2, a de-coherence between the cingulate and insula cortex as well as the hippocampal formation and periaqueductal gray. Although, structures such as the insula are part of the task positive network, it is difficult to say whether this particular connectivity pattern has to do with the task positive network or connectivity uniquely related to an anxious state of mind. In order to more accurately demonstrate if awake rodents possess a task positive network, an experimental paradigm involving visual processing in a population of habituated rats could be performed. It is noteworthy that sustained illumination is known to induce anxiety in rodents [36]. Therefore, the duration of the visual stimulation must be taken into careful consideration.

This investigation demonstrates that the anterior cingulate is an important component for both the DMN-like and anxiety networks, and whose connectivity behavior is modulated during the habituation process. Anterior cingulate-based functional connectivity dynamics occurring within DMN-like and anxiety networks were not only observed between Days 2-8 as habituation ensued. Given the integrative type role of the anterior cingulate cortex in mediating a range of CNS functions such processing negative affect [9], shifts in mood and attention [37] or monitoring autonomic function [38], this particular limbic structure appears to be well suited as a key component during the transition between DMN-like and anxiety networks or vice versa. From a structural connectivity perspective, the anterior cingulate cortex has efferent and afferent connections to a range of CNS structures that play a role in both the default mode and anxiety networks [18], [39], [40]. Thus, the changeover between the two networks occurs in part as a result of anterior cingulate activity and its direct interaction with other DMN and anxiety network structures. Nonetheless, the activity and connectivity of other CNS structures, such as the reticular formation should also be considered given the key role this structure has in mediating alertness or sensory gating [41].

Resting-state fMRI data were collected in awake, non-anesthetized rats, as anesthesia has been shown to alter functional connectivity [6], [8] and brain hemodynamics [42]. However, awake imaging methodology is limited by increased incidence of motion that can render a dataset unusable. Another point of consideration is that in awake rodent imaging, even following habituation, physiological conditions might not truly reach baseline levels. For example, in the present study, group-level respiratory rates at Day 8 were measured to be ∼125 breaths/minute, while baseline respiratory rates in awake rats may range between 70-115 breaths/minute (www.criver.com). Nonetheless, procedures such as regression of white matter and CSF timecourses from functional connectivity analysis can be implemented to help mitigate global physiological effects. Moreover, as this study demonstrates, the CNS may be in different functional states (i.e., anxious vs. DMN-like) depending on how well acclimated an awake animal is to the MRI scanner environment. In awake imaging studies where a pharmacological effect on CNS circuitry might be characterized, the degree to which subjects are acclimated or habituated is important to consider as this may have impact on pharmacodynamic measures.

### Study Limitations

#### Habituation vs. Learned Helplessness

The emergence of DMN-like activity as well as the lower respiratory rate at Day 8 is likely to have resulted from a habituation or acclimation process to a novel MRI environment. Observations such as the inverse-correlation between cingulate-anteroventral thalamus functional connectivity and head displacement are in accord with this viewpoint. However, it is also possible that the awake rodents may have been in a state of learned helplessness or chronic stress at Day 8. The presence of learned helplessness or chronic stress could underlie observation such as reduced head displacement, which could also affect functional connectivity results. To adequately determine whether rodents undergoing multiple awake scanning sessions do in fact experience habituation rather than a learned helplessness or chronic stress, additional behavioral procedures could be carried out. For example, a comparison of each subjects' ability to escape adverse situations after each scanning session could be assessed. Future work could also involve a forced swim test before scanning on Day 8, where the presence of learned helplessness through this particular procedure may prohibit DMN-like activity. Moreover, an assessment of neuropharmacology in rodents at Day 8 of scanning may help elucidate the presence of learned helplessness or chronic stress [43], [44].

#### DMN and Medial Prefrontal Cortex

In the current study, the DMN-like functional connectivity pattern observed in rodents consisted of CNS structures such as the anterior cingulate, retrosplenial, parietal and hippocampus, but did not include the medial prefrontal cortex. Specifically, mixed-effects functional connectivity contrast analysis (Day 2 vs Day 8) did not yield significant and enhanced functional connectivity between the anterior cingulate and medical prefrontal cortices at Day 8. This observation is divergent from the DMN previously identified and characterized in humans and non-human primates [1], [2], [3], [4], [5], where the medial prefrontal cortex shows significant functional connectivity with other DMN structures. This unexpected difference in DMN or DMN-like functional connectivity patterns between humans and non-human primates versus rodents cannot be fully explained by current dataset. However, it is speculated that factors such as slightly higher stress levels (as indicated by respiratory rates at Day 8) compared to baseline may play a role in the discrepant finding. This is particularly true given the known function of the medial prefrontal cortex in stress or anxiety [45]. The lack of functional connectivity between medial prefrontal regions and DMN regions such as the retrosplenial cortex in the habituated rodent is not a finding unique to this investigation. In recent work by Liang and colleagues, the medial prefrontal cortex was observed to be functionally connected to regions such as the caudate-putamen, nucleus accumbens, insula, anterior cingulate, hypothalamus, hippocampus, anterior olfactory nucleus and motor cortex [21]; a network which is very different from the human and non-human primate DMN.

#### Training and Scanning Duration

The optimal number of resting-state fMRI scanning sessions needed in order to measure significant DMN-like functional connectivity within awake rodents remains unknown. Specifically, it is unknown whether it is best to perform multiple consecutive scanning sessions or multiple scanning sessions interleaved with ‘rest’ days. It may prove true that awake, resting-state fMRI performed for shorter periods of time (e.g., t<30 mins), but with higher frequency could be less stressful for the subjects and habituation could be achieved sooner. More acclimation within the MRI simulator prior to actual resting-state fMRI scanning may also be beneficial with regards to the habituation process. Although the training and scanning protocol implemented here is similar to past awake rodent fMRI studies [11], [20], [21], a systematic comparison of distinct training and scanning protocols along with measurement of global physiology are necessary.

#### Measurement of Corticosterone Levels

A general limitation of rodent fMRI studies similar to the current investigation is the inability to obtain blood or plasma samples during the actual fMRI scan. This is simply due to the positioning of the animal within the center of the MRI bore; thus making parts of the animal (e.g., tail vein) inaccessible. Moreover, if blood or plasma samples are obtained subsequent to the scanning session, the animal is often first handled, moved out of the MRI scanner to a different environment and then anesthetized. Such perturbations more than likely confound measurements of corticosterone levels, and also make it difficult or inaccurate to relate fMRI-based measures with corticosternone levels. In fact, in the current study, the presence of isoflurane as well as handing of the animals may have caused the lack of significant change in corticosterone levels during the initial handling and training period. In future studies, it would be advantageous to at least sample corticosterone levels in the non-anesthetized state.

In conclusion, this investigation revealed DMN-like connectivity in awake, habituated rodents, while anxiety network connectivity was measured in the same animals during the awake, non-habituated state. In characterizing these two networks during the habituation process, the anterior cingulate was observed to be an important component. Future work similar to this study as well as that performed by Liang et al. [21], will further determine the functional organization of the rodent brain as well as the functional role of various CNS circuits. Moreover, how rodent CNS circuits are modulated pharmacologically, by an induced diseased state or both may also help to address the important issue of functional properties of CNS circuits and the utility of their characterization.
